# Engineering a Compartmentalized Multi-Cell Co-Culture Hydrogel System Using Beeswax/Fucoidan/Alginate for Cultured Meat Modeling

**DOI:** 10.3390/foods15101715

**Published:** 2026-05-13

**Authors:** Jihad Kamel, Jun-Yeong Lee, Sadia Afrin, Usha Yadav, Chandra Jit Yadav, Sung Soo Han, Kyung-Mee Park

**Affiliations:** 1 College of Veterinary Medicine, Chungbuk National University, Cheongju 28644, Republic of Korea; jihadshabaan@gmail.com (J.K.); dkujunyeong@naver.com (J.-Y.L.); sadiaafrin1406@gmail.com (S.A.); ushacj.23@gmail.com (U.Y.); chandrajityadav84@gmail.com (C.J.Y.); 2 School of Chemical Engineering, Yeungnam University, 280 Daehak-ro, Gyeongsan 38541, Republic of Korea; sshan@yu.ac.kr

**Keywords:** alginate hydrogel, beeswax, C2C12 myoblasts, fucoidan, fibroblast, indirect co-culture

## Abstract

**Objective**: Developing foundational biomaterial platforms for cultured meat research requires 3D co-culture systems capable of supporting multiple relevant cell types in a spatially organized manner. This study aimed to establish a compartmentalized tri-culture hydrogel disc incorporating a lipid-containing barrier phase as a proof-of-concept in vitro model. **Methods**: Beeswax/alginate (Bw/Algi) hydrogels were fabricated and evaluated for morphology and cytocompatibility as a lipid-containing scaffold component. Fucoidan/alginate (Fu/Algi) hydrogels were prepared at varying fucoidan concentrations and screened to identify conditions compatible with C2C12 viability and early-stage differentiation. A composite beeswax/fucoidan/alginate disc (Bw/Fu/Algi) was then assembled by casting cell-laden Fu/Algi regions (myoblasts, fibroblasts, and endothelial cells), separated by Bw/Algi barrier layers and ionically crosslinked with CaCl_2_. Scaffold performance was assessed using standard assays for morphology, cytocompatibility, myogenic marker expression, protein production, and thermal stability. **Results**: Bw/Algi supported cytocompatible C2C12 attachment and growth, while Fu/Algi exhibited concentration-dependent effects on myogenic marker expression, enabling selection of an optimized fucoidan concentration for 3D assembly. The final Bw/Fu/Algi disc maintained viable compartmentalized tri-culture and supported indirect co-culture through spatial separation by the Bw barrier. Myogenic regions exhibited myogenic marker expression with measurable protein production, and differential scanning calorimetry confirmed structural stability under heating. **Conclusion**: This work establishes a Bw/Fu/Algi tri-culture disc integrating a lipid-containing barrier component with hydrogel-based myogenic compartments, providing a preliminary platform for multicellular in vitro modeling and scaffold design relevant to cultured meat research.

## 1. Introduction

Engineering structured skeletal muscle tissue in vitro requires scaffold systems that promote organized myogenic development rather than dispersed cell growth [1,2,3,4,5]. However, achieving aligned and mature muscle architecture remains challenging. In native muscle, myogenesis depends on coordinated interactions between myogenic, stromal, and endothelial cells within the ECM microenvironment [6,7,8,9]. These interactions provide biochemical signals and local regulation governing the proliferation, fusion, and maturation of myoblasts. Accordingly, in vitro models must support cell viability and differentiation while enabling reproducible 3D culture under diffusion and spatial constraints [8,9]. Conventional monoculture systems fail to recapitulate the complex signaling interactions among multiple cell types, and robust myogenic differentiation within 3D culture systems remains difficult to achieve. [10,11,12,13].

Indirect co-culture enables paracrine signaling through soluble factor exchange without physical mixing of cell populations, thereby reducing direct competition and overgrowth [14,15,16,17]. This approach improves experimental controllability and minimizes disruption of myogenic differentiation by rapidly proliferating support cells [18]. Fibroblasts can modulate ECM remodeling and growth factor availability, while endothelial cells contribute pro-angiogenic signaling that supports cell survival and differentiation [4,11,19]. Incorporating these supportive populations into engineered systems may therefore improve myogenic outcomes compared with myoblast monocultures [13].

Successful implementation of indirect multicellular culture requires scaffold systems that simultaneously maintain cellular viability, diffusion capacity, and structural stability to support myotube formation. [18,20,21,22]. A trade-off persists: mechanically stable scaffolds may reduce bio-permissiveness, whereas highly hydrated hydrogels often lack durability in 3D constructs [23,24]. Alginate (Algi) is widely used due to its mild gelation and biocompatibility; however, its limited intrinsic cell adhesion can restrict myoblast attachment and maturation without additional bioactive cues [25,26,27]. Accordingly, composite strategies have been explored to improve alginate-based scaffolds while retaining their fabrication advantages [28,29]. Fucoidan (Fu), a brown seaweed-derived sulfated polysaccharide, has been investigated as a bioactive hydrogel additive with regenerative potential [30,31,32]. Importantly, Fu concentration can influence both hydrogel properties and cellular responses [30]. In parallel, beeswax (Bw) has been incorporated into composite biomaterials to enhance hydrophobicity and mechanical stability. Bw-containing membranes have also been explored as barrier-forming phases that modulate cell attachment and spatial organization [33,34,35,36,37,38,39,40,41]. Incorporating a barrier element into a hydrogel platform may therefore enable physical separation of distinct cell populations while preserving indirect paracrine communication [33,34,35].

Although previous hydrogel systems have improved scaffold composition and cytocompatibility, maintaining spatially separated multi-cell-type organization within a single construct remains insufficiently explored [9,42,43].

To address limitations associated with conventional hydrogels, our previous studies focused on developing plant-based scaffold platforms with improved physicochemical and biological performance. Protein- and starch-enriched alginate systems were designed to enhance mechanical properties and metabolic similarity [42], while alginate–zein composites incorporating cell-derived components were developed to promote biochemical signaling and myotube alignment [5]. However, these approaches were based on homogeneous scaffold architecture and did not support the co-culture of multiple cell types within a single construct. Therefore, the present study aimed to establish a compartmentalized Bw/Fu/Algi hydrogel integrating a lipid-containing barrier phase with cell-laden regions to support multicellular co-culture. We hypothesized that this configuration would facilitate organization of myoblasts, fibroblasts, and endothelial cells while supporting early-stage myogenic development within a three-dimensional culture environment.

## 2. Materials and Methods

### 2.1. Cell Culture

Mouse C2C12 myoblasts (CRL-1772, ATCC, Manassas, VA, USA), mouse embryonic fibroblasts (MEF-1; CRL-2214, ATCC, Manassas, VA, USA), and EA.hy926 human endothelial cells (HECs) (ATCC, Manassas, VA, USA) were co-cultured under both 2D and 3D conditions. Cells were maintained in high-glucose DMEM supplemented with 2 mM L-glutamine (Thermo Fisher Scientific, Waltham, MA, USA), 10% (*v*/*v*) fetal bovine serum (FBS; Cytiva, Marlborough, MA, USA), and 1% (*v*/*v*) antibiotic–antimycotic solution (ABAM; WelGene, Gyeongsan, Republic of Korea), and incubated at 37 °C in a humidified atmosphere containing 5% CO_2_.

### 2.2. Biocomposite Bw/Algi Hydrogel

#### 2.2.1. Preparation of the Hydrogel

The beeswax/alginate (Bw/Algi) composite hydrogel was prepared as a lipid–polysaccharide scaffold intended to function as a barrier phase. Briefly, sodium alginate (1% *w*/*v*; Sigma-Aldrich, St. Louis, MO, USA) was dissolved in distilled water (DW) under continuous magnetic stirring at 50 °C until a homogeneous solution was obtained. In parallel, refined beeswax (10% *w*/*v*; Sigma-Aldrich) and coconut oil (0.5% *w*/*v*) were heated to 70–80 °C until fully melted. The molten lipid phase was then gradually introduced into the alginate solution under continuous stirring to form a uniform emulsion. Mixing was maintained at an elevated temperature to prevent premature solidification of beeswax. The resulting viscous mixture was cast into the concentric mold or a one-well culture plate and allowed to stabilize at room temperature. Gelation was subsequently achieved by adding 2% (*w*/*v*) CaCl_2_ solution for 15–20 min to induce ionic crosslinking. The formed hydrogel was rinsed with phosphate-buffered saline (PBS) to remove excess calcium ions prior to further use.

#### 2.2.2. Physical Characteristics

Samples were prepared by casting 10 mL of the hydrogel solution into a 10 cm culture dish, followed by ionic crosslinking using 2% (*w*/*v*) CaCl_2_ solution. After gelation, cylindrical samples were obtained using a 6 mm biopsy punch. The thickness of each sample was measured using a digital caliper and maintained at approximately 7 mm.

Hydrogel swelling behavior was assessed under incubation at 37 °C and 5% CO_2_ to replicate standard cell culture conditions and maintain physiological pH via the bicarbonate buffering system, ensuring consistency with subsequent cell-based experiments. Prior to testing, scaffolds were freeze-dried at −50 °C for 24 h, and the initial dry weight (*W*_0_) was recorded as the baseline.

Samples were then immersed in fresh culture medium and collected at predefined time points (0, 1, 3, 5, and 7 days), with the medium refreshed every two days. At each interval, independent samples were used (*n* = 5 per time point), and excess surface moisture was gently removed by blotting prior to measuring the wet weight (*W_t_*). A destructive sampling approach was employed, where each sample was measured only once at its designated time point. The swelling ratio (SR) was calculated using the following equation [44]:

(1) *SR *(%) = (*W_t_* − *W*_0_)/*W*_0_ × 100

To ensure independent evaluation of degradation, a separate set of scaffolds was used. For degradation analysis, the initial dry mass (*W*_0_) was determined as described above. At each time point, samples were retrieved from the medium, dried at 60 °C for 4 h, and the remaining dry mass (*W*) was measured [45]. The degradation rate was calculated as:

(2) *Degradation rate* (%) = (*W*_0_ − *W*)/*W*_0_ × 100

Each hydrogel scaffold was treated as an individual experimental unit. Swelling and degradation tests were performed using eight independent samples per group at each time point, following a destructive sampling approach.

Two independent 3D co-culture hydrogel discs were fabricated. Following proliferation (day 3) and differentiation (day 7), the Bw/Algi barrier was mechanically displaced using a 6 mm biopsy punch, and the constructs were cross-sectioned (*n* = 4). Sections were obtained from both the central region (separating HECs and MEFs) and the peripheral region (separating MEFs and C2C12 cells). Each scaffold was stained with DAPI and imaged at four distinct fields per sample.

#### 2.2.3. Cytotoxicity

Indirect cytotoxicity of the Bw/Algi hydrogel was evaluated using a CCK-8 assay (Sigma-Aldrich) at 1, 3, and 7 days. C2C12 cells were exposed to Bw/Algi-conditioned media, and cell viability was quantified according to the manufacturer’s instructions. Absorbance was measured at 450 nm using a NanoQuant plate reader (TECAN Ltd., Männedorf, Switzerland).

Cell viability was further assessed by Live/Dead staining using a Live/Dead™ Cell Imaging Kit (Thermo Fisher Scientific). Fluorescence images were acquired using an inverted fluorescence microscope (OPTINITY, KI-2000F, Korea Lab Tech, Hwaseong-si, Republic of Korea). The positive control consisted of 2D cell culture without Bw/Algi, while the negative control comprised fresh medium.

#### 2.2.4. Cell Seeding

Bw/Algi hydrogels were cast in a 35 mm confocal dish (SPL, Gyeonggi, Republic of Korea), and circular scaffolds were obtained using a 6 mm biopsy punch. The scaffold thickness was adjusted to 1 mm and confirmed using a digital caliper. The punched scaffolds were transferred to a 35 mm, 4-compartment confocal dish, and C2C12 cells were seeded onto the scaffold surface at a density of 5 × 10^4^ cells per scaffold. Cells were cultured in DMEM supplemented with 10% FBS and 1% ABAM. Cell attachment was assessed using scanning electron microscopy (SEM) at day 1. Cell viability and proliferation (day 3) were assessed by DAPI nuclear staining (1:1000; Sigma-Aldrich) and Live/Dead™. Bw-only hydrogels served as controls.

#### 2.2.5. Scanning Electron Microscopy (SEM)

Samples were first gently washed with PBS to remove non-adherent cells and then fixed in 2.5% glutaraldehyde prepared in 0.1 M phosphate buffer (pH 7.4) at 4 °C for 1 h. Following fixation, samples were washed twice with PBS for 10 min each. Post-fixation was performed using 1% osmium tetroxide (OsO_4_) in 0.1 M phosphate buffer for 30 min. The samples were then washed again with PBS for 10 min. Dehydration was carried out through a graded ethanol series (30%, 50%, 60%, 70%, 80%, 90%, 95%, and 100%), with each step performed for 10 min, followed by two additional washes in 100% ethanol for 10 min each.

Subsequently, samples were treated with isoamyl acetate for 10 min (two times) and allowed to dry completely under a fume hood for 24 h. The dried samples were mounted onto aluminum stubs and sputter-coated with platinum (Pt) or gold (Au) to enhance conductivity. The surface morphology and pore structure of Bw/Algi were examined and compared with those of Bw. SEM observation was performed using a Gemini 560 microscope (Carl Zeiss, Oberkochen, Germany). Images were acquired at appropriate magnifications for pore structure and cell attachment.

Pore structure analysis was performed using ImageJ software (version 1.47) (National Institutes of Health, Bethesda, MD, USA). SEM images were converted to 8-bit grayscale and thresholded to distinguish pore regions from the hydrogel matrix. The threshold was applied consistently across all images to ensure uniform analysis. Following binarization, the total pore area was quantified, and the pore area percentage was calculated as the ratio of pore area to the total image area. Multiple SEM images from different regions of each sample were analyzed (*n* = 3) to obtain representative values. SEM images for pore morphology and cell attachment were obtained at magnifications corresponding to 2 µm and 10 µm scale bars, respectively.

#### 2.2.6. Fourier Transform Infrared Spectrometer (FTIR)

FTIR was used to analyze the functional groups of Bw within the Bw/Algi hydrogel. Spectra were acquired using an FTIR spectrometer (Cary 670 main bench coupled with Cary 620 microscope; Agilent Technologies, Santa Clara, CA, USA) over a wavenumber range of 500–4500 cm^−1^ at a spectral resolution of 4 cm^−1^.

### 2.3. 2D Indirect Co-Culture of C2C12 Myoblasts, MEF, and HECs

A 2D indirect co-culture system was established using a 4-well confocal dish. C2C12, MEFs, and HECs were individually seeded at a density of 3 × 10^4^ cells per well and allowed to attach for 1 h. To create interconnected compartments for cell co-culture, openings were made in the well walls using a heat-treated 18-gauge needle. This method enabled the exchange of conditioned media among the different cell types.

The experiment was conducted in five individual dishes:

(1) C2C12 cultured alone (control)
(2) C2C12 co-cultured with MEFs
(3) C2C12 co-cultured with HECs
(4) C2C12 co-cultured with MEFs and HECs
(5) C2C12 co-cultured with MEFs, HECs, and Bw/Algi (quadrate co-culture system)

Cells were maintained under shared culture conditions during the proliferation phase, followed by differentiation using differentiation medium containing 2% horse serum (HS) and 1% ABAM. Differentiation was performed for 7–10 days, with medium replaced every two days.

### 2.4. Optimization of Fucoidan (Fu) Concentration

The Fu/Algi formulation was intended to serve as the main cell-laden hydrogel in this study. To determine an appropriate concentration for the hydrogel, Fu (Sigma-Aldrich) was incorporated into 1% (*w*/*v*) Algi at final concentrations of 0, 1, 20, 30, and 50 µg/mL. The 0 µg/mL group (Algi only) served as the control. Hydrogel formulations were cast in 4-well culture plates, and cells were seeded at a density of 5 × 10^4^ cells per well. The Fu/Algi hydrogels were ionically crosslinked using 2% (*w*/*v*) CaCl_2_.

Cytocompatibility was evaluated during the proliferation period using a CCK-8 assay on day 7 and Live/Dead staining at days 1, 3, and 7. Differentiation medium was then applied and maintained for 7 days. Microscopy images were acquired to quantify myotube formation, and myotube analysis was performed using ImageJ software.

### 2.5. Fabrication of the Bw/Fu/Algi Hydrogel Disc

After successfully implementing the 2D quadrate co-culture system, Fu (20 µg/mL) was incorporated into a 1% Algi solution to prepare a 3D Fu/Algi hydrogel. A stainless-steel circular mold placed in a 10 cm dish was used to partition the hydrogel into five concentric chambers. Bw/Algi functioned as a lipid-based barrier between the Fu/Algi compartments. The hydrogel layers were organized from the periphery to the center as follows:

(1) 6 mL Fu/Algi containing 2.5 × 10^5^ C2C12 cells.
(2) 6 mL Bw/Algi.
(3) 4 mL Fu/Algi containing 2.5 × 10^5^ MEF cells.
(4) 6 mL Bw/Algi; and
(5) 2 mL Fu/Algi containing 2.5 × 10^5^ HECs.

Subsequently, 2% CaCl_2_ was added to cover all chambers for 15–20 min to crosslink the hydrogel. The CaCl_2_ solution was replaced once with PBS, and growth medium was added for 3 days to allow proliferation, followed by differentiation medium for 7–10 days with medium changes every 2 days.

The 3D hydrogel disc was fabricated using a concentric circular mold composed of radially arranged compartments with increasing diameters from the center toward the periphery. This design inherently resulted in different hydrogel volumes across compartments. Consequently, although equal cell numbers were seeded, local cell density varied according to compartment volume.

### 2.6. Immunofluorescence Staining

C2C12 differentiation was assessed by immunofluorescence staining for desmin and myosin. Hydrogels were washed with PBS, fixed in BIOFIX HD (BIOGNOST, Zagreb, Croatia) for 20 min, permeabilized with 0.2% Triton X-100 (SAMCHUN, Ulsan, Republic of Korea) for 10 min, and washed with PBST. Non-specific binding was blocked with 1% bovine serum albumin (BSA; Sigma-Aldrich) in 1× PBST for 30 min.

Samples were incubated with primary antibodies against PAX-7 (1:100, Invitrogen, Waltham, MA, USA), α-desmin (1:100, Sigma-Aldrich), and skeletal muscle myosin (Sigma-Aldrich) for 1 h at room temperature, followed by three washes with PBST. Secondary antibody incubation was performed using goat anti-mouse IgG (1:500, Invitrogen) for 1 h. Nuclei were counterstained with DAPI (1:1000) for 5 min. Myotube area, diameter, alignment, and fusion index were quantified from desmin-stained images using ImageJ software (version 1.47). The relative frequency (%) of myotube orientation was determined using the OrientationJ plugin (Version 2.0.7) [46].

(3) Fusion index % (FI) = (the number of nuclei in myotube of 2 myonuclei/the total number of nuclei) × 100

### 2.7. Quantitative PCR (qPCR)

Total RNA was isolated using the RNeasy Mini Kit (Qiagen, Hilden, Germany) and quantified by spectrophotometry. Complementary DNA (cDNA) was synthesized using TOPscript™ RT DryMIX (dN6 Plus) (Enzynomics, Daejeon, Republic of Korea) according to the manufacturer’s instructions. Quantitative PCR was performed using gene-specific primers for GAPDH, Cdk1, and myogenin (Table 1). Relative gene expression was calculated using the ΔΔCt method, with GAPDH as the reference gene. β-actin was additionally used as a secondary housekeeping gene to confirm normalization consistency. C2C12 cells cultured under monoculture conditions served as the control group.

### 2.8. Protein Content Evaluation

Protein content produced by C2C12 cells in the 2D quadrate co-culture system, 3D hydrogels, and mouse muscle was evaluated. Following 10 days of differentiation, all samples were freeze-dried for 24 h, and an equivalent dry weight was used for analysis. Dried samples were lysed in 1 mL RIPA buffer (Sigma-Aldrich) at 4 °C for 30 min, followed by centrifugation at 2700× *g* for 10 min. Protein concentration in the supernatant was quantified using a bicinchoninic acid (BCA) assay kit (iNtRON Biotechnology, Gyeonggi, Republic of Korea). All experiments were performed independently in triplicate.

### 2.9. Thermal Properties of the 3D Scaffold

The thermal properties of the 3D hydrogel scaffolds were evaluated and compared with native meat using differential scanning calorimetry (DSC). DSC analysis was performed using a Q2000 instrument (TA Instruments, New Castle, DE, USA). Samples (10–20 mg) were sealed in 40 µL aluminum pans (PerkinElmer Inc., Waltham, MA, USA) and heated from 40 °C to 180 °C at a rate of 50 °C/min. This heating rate was applied consistently across all samples for comparative analysis.

### 2.10. Statistical Analysis

All data are presented as mean ± standard error (SE). Statistical analyses were performed using GraphPad Prism version 8.0.1.244 (64-bit, Windows). For multi–time point analyses, two-way ANOVA was used, followed by Holm–Šidák multiple comparisons test where appropriate. Single time-point comparisons were analyzed using one-way ANOVA followed by Tukey’s multiple comparisons test. A *p*-value < 0.05 was considered statistically significant.

## 3. Results

### 3.1. Physical and Chemical Properties of Bw/Algi Hydrogel

**Figure 1:**
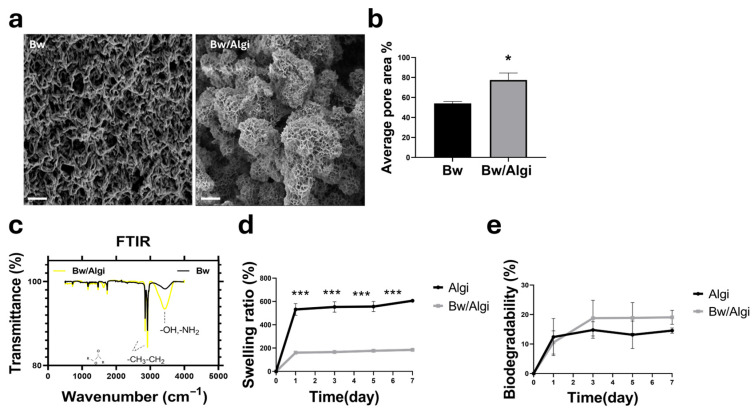
Surface morphology and physical properties of Bw/Algi hydrogels. (**a**,**b**) SEM images and quantitative pore area analysis of beeswax (Bw) and beeswax/alginate (Bw/Algi) hydrogels (*n* = 3). Scale bar: 2 µm. (**c**) FTIR spectra showing characteristic functional groups of Bw and Bw/Algi hydrogels. (**d**,**e**) Swelling and biodegradability of hydrogels were measured over 7 days (*n* = 5). Data are presented as mean ± SE. Statistical significance was determined using one-way ANOVA followed by Tukey’s multiple comparisons test, and two-way ANOVA followed by Holm–Šidák correction, as appropriate; * *p* < 0.05, *** *p* < 0.001.

SEM analysis showed that Bw/Algi hydrogels had a higher pore area percentage (77.57% ± 7.88) than Bw alone (54.16% ± 4.64), indicating increased structural porosity following alginate incorporation (Figure 1a,b). FTIR spectra confirmed the presence of Bw-associated functional groups in the Bw/Algi hydrogel, including ester, hydrocarbon, and hydroxyl-related bands, demonstrating retention of key chemical features after blending (Figure 1c).

Swelling and biodegradability were evaluated over 7 days. Algi showed a higher swelling ratio than Bw/Algi, reaching 606.54% ± 13 compared with 185.01% ± 22, indicating reduced water uptake in the composite hydrogel (Figure 1d). No significant difference in biodegradability was observed during the same period, with values of 14.57% ± 2.03 for Algi and 19.06% ± 1.20 for Bw/Algi, suggesting comparable degradation behavior (Figure 1e).

The Bw/Algi barrier exhibited biodegradability within the 3D hydrogel disc during both proliferation and differentiation periods (Figure S1a,b). These findings indicate that alginate incorporation modified the structural and physicochemical behavior of the Bw-based hydrogel while preserving key material-associated functional groups.

### 3.2. Cytotoxicity and Cell Attachment Through Bw/Algi Hydrogel

**Figure 2:**
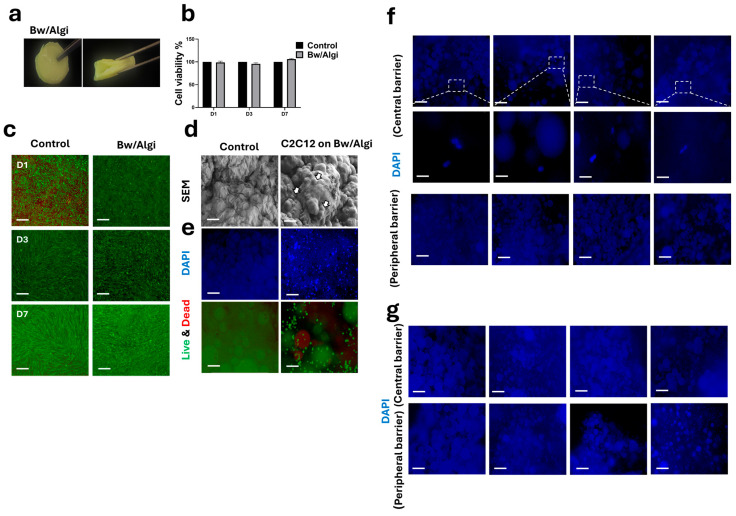
Cytotoxicity and cell attachment of C2C12 myoblasts through the Bw/Algi hydrogel. (**a**) Macroscopic appearance of the beeswax/alginate (Bw/Algi) hydrogel showing flexibility under compression. (**b**,**c**) Cytotoxicity assessment of Bw/Algi by CCK-8 assay and Live/Dead staining after indirect co-culture (*n* = 4). The control group consisted of C2C12 cells cultured without Bw/Algi. Live cells are shown in green and dead cells in red. (**d**) SEM images showing C2C12 cell attachment on the surface of Bw/Algi hydrogels after 1 day of culture. White arrows indicate attached cells. Scale bar: 10 µm. (**e**) DAPI and Live/Dead staining showing C2C12 cells on Bw/Algi hydrogels after 3 days of culture. Scale bar: 200 µm. 3D Bw/Fu/Algi hydrogel discs were analyzed after proliferation/differentiation. Sections of Bw/Algi were obtained from both the central region (separating HECs and MEFs) and the peripheral region (separating MEFs and C2C12 cells). (**f**) After 3 days of proliferation, limited cell presence was observed at the central barrier, while no visible cells were observed at the peripheral barrier. The dashed box indicates the area shown at higher magnification. (**g**) After 7 days of differentiation, no visible cells were detected at either the central or peripheral Bw/Algi barrier. Scale bars = 200 and 100 μm. Data are presented as mean ± SE. Statistical analysis was performed using two-way ANOVA followed by Holm–Šidák multiple comparisons test; no significant differences were detected.

C2C12 cells were cultured in indirect contact with Bw/Algi hydrogels for 3–7 days. The Bw/Algi hydrogel exhibited flexibility under manual compression (Figure 2a). Indirect co-culture assays showed no significant cytotoxicity over 7 days, as indicated by Live/Dead staining and CCK-8 results (Figure 2b,c). Cells cultured without Bw/Algi served as a control.

For direct contact evaluation, C2C12 cells were seeded onto the surface of Bw/Algi hydrogels and cultured for up to 3 days. SEM analysis confirmed cell attachment after 1 day (Figure 2d). By day 3, cell presence and proliferation on the hydrogel surface were observed using DAPI and Live/Dead staining (Figure 2e).

Two independent 3D Bw/Fu/Algi hydrogel constructs were analyzed following proliferation/differentiation. Sections corresponding to the Bw/Algi barrier were obtained from the central interface (between HECs and MEFs) and the peripheral interface (between MEFs and C2C12). Compared with the scaffold control (free-cell condition) (Figure 2e), DAPI staining showed a limited number of cells at the central barrier, whereas no visible cells were detected at the peripheral barrier (Figure 2f,g).

Together, these findings showed that Bw/Algi hydrogels maintain cytocompatibility and support initial cell attachment and maintain compartmental organization across barrier regions.

### 3.3. 2D Indirect Compartmentalized Co-Culture System

**Figure 3:**
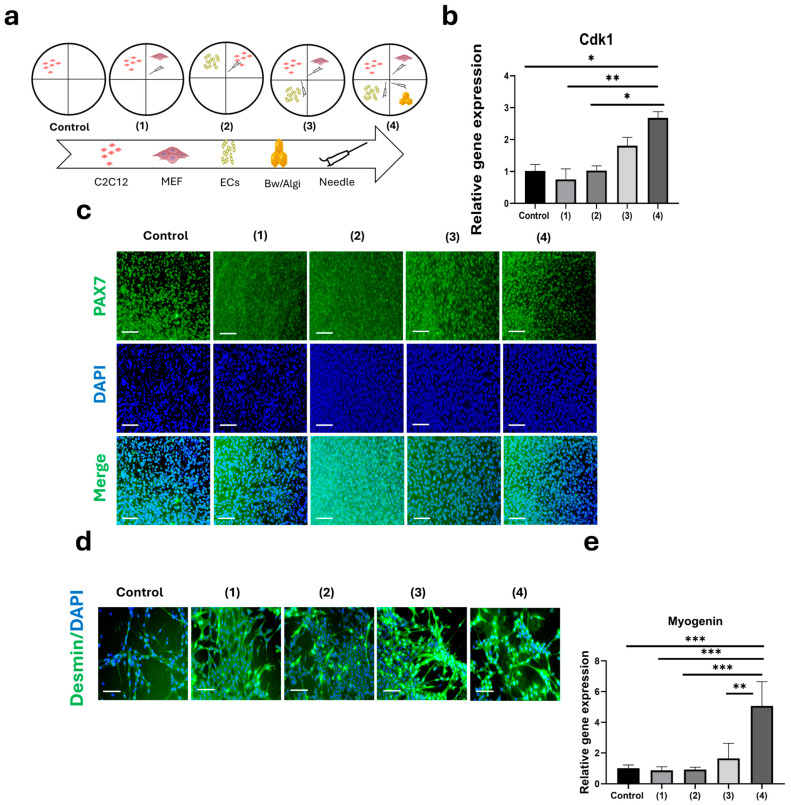
Quadrant indirect co-culture of C2C12 with MEF, HECs, and Bw/Algi. (**a**) Schematic of the 2D co-culture conditions: (1) MEF, (2) HECs, (3) MEF + HECs, and (4) MEF + HECs + Bw/Algi. (**b**) Cdk1 mRNA expression in C2C12 cells measured on day 3. (**c**) PAX7 immunostaining of C2C12 cells during the proliferation phase (up to day 7). Scale bar: 100 µm. (**d**) Desmin immunofluorescence in C2C12 cells after 7 days of differentiation in the co-culture system. Scale bar: 100 µm. (**e**) Relative mRNA expression of the late myogenic differentiation marker myogenin after 7 days of differentiation. The control group represents C2C12 monoculture. Data are presented as mean ± SE (*n* = 4). Statistical significance was assessed using one-way ANOVA followed by Tukey’s multiple comparisons test; * *p* < 0.05, ** *p* < 0.01, and *** *p* < 0.001.

C2C12 cells were indirectly co-cultured with MEFs, HECs, and Bw/Algi using a four-compartment culture plate, as illustrated in the schematic design (groups 1–4) (Figure 3a). This system allowed physical separation of cell populations while permitting exchange of soluble factors through shared culture media. On day 3, Cdk1 (a key regulator of cell-cycle progression) was significantly increased in group 4 compared with several other conditions (Figure 3b). No significant difference was observed between groups 3 and 4. PAX7 immunostaining was detected in C2C12 cultures under all conditions up to day 7 (Figure 3c). Overall, group 4 included Bw, indicating that Bw incorporation did not alter C2C12 proliferative activity.

Following differentiation induction, early-stage myogenic differentiation was evaluated (Figure 3d). Desmin immunostaining was observed in multiple co-culture groups (1, 3, and 4), whereas weaker staining was detected in the C2C12 monoculture (control), reflecting differences in desmin expression among groups. Consistent with these findings, myogenin gene expression was significantly upregulated in group 4 compared with all other conditions (Figure 3e).

Collectively, these results showed that the tri-co-culture configuration influenced C2C12 proliferative activity and myogenic marker expression.

### 3.4. Effect of Fu Concentration on C2C12 Growth and Differentiation

**Figure 4:**
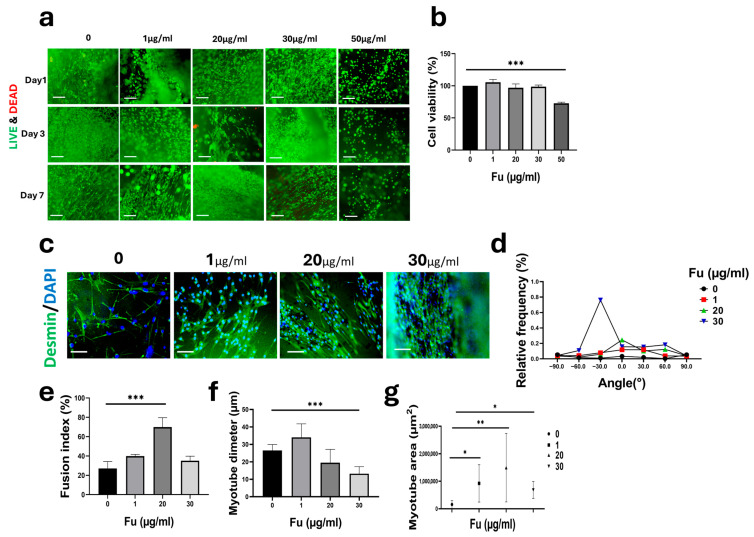
Effect of fucoidan (Fu) concentration on proliferation and myogenic differentiation of C2C12 cells in Fu/Algi hydrogels. (**a**,**b**) Cell viability and proliferation were assessed by Live/Dead staining and CCK-8 assay after 7 days (*n* = 4). Scale bar: 200 µm. (**c**) Immunofluorescence staining of desmin after 7 days of differentiation. Scale bar: 100 µm. (**d**) Myotube alignment analysis showing the highest alignment frequency at 0° in the 20 µg/mL group compared to the control (0) (*n* = 3). (**e**) Quantitative analysis of fusion index, (**f**) myotube diameter, and (**g**) myotube area quantified from desmin-positive structures using ImageJ software. (*n* = 3). Data are presented as mean ± SE. Statistical significance was assessed using one-way ANOVA; * *p* < 0.05, ** *p* < 0.01, *** *p* < 0.001.

Fu was incorporated into Algi hydrogels at concentrations of 1, 20, 30, and 50 µg/mL. Live/Dead staining showed maintained cell viability over 7 days. At Fu concentrations up to 30 µg/mL, cells displayed a spindle-shaped morphology, consistent with normal attachment and spreading (Figure 4a). CCK-8 analysis confirmed sustained cell proliferation over 7 days. Proliferation was higher at concentrations up to 30 µg/mL than in the Algi-only control (Figure 4b). In contrast, the 50 µg/mL group showed significantly reduced proliferation, indicating lower cytocompatibility at this condition.

Early myogenic differentiation was assessed by desmin immunofluorescence after 7 days of induction (Figure 4c), across all concentrations. Myotube alignment (desmin^+^ myotubes) was analyzed over an angular range of −90° to 90°, with 0° defined as the primary orientation endpoint. The 20 µg/mL group showed a 7.34-fold increase in alignment at 0° compared with the control (Figure 4d). A significant increase in the myotube fusion index was also observed at 20 µg/mL (Figure 4e). Quantitative image analysis showed no significant difference at 20 µg/mL in myotube diameter, whereas a significant increase was detected in myotube area (Figure 4f,g), indicating differences in myotube coverage among groups.

Overall, the highest alignment, fusion index, and myotube area were observed at 20 µg/mL. Based on these results, 20 µg/mL Fu was selected for subsequent 3D culture experiments.

### 3.5. Bw/Fu/Algi 3D Tri-Culture Hydrogel Disc

**Figure 5:**
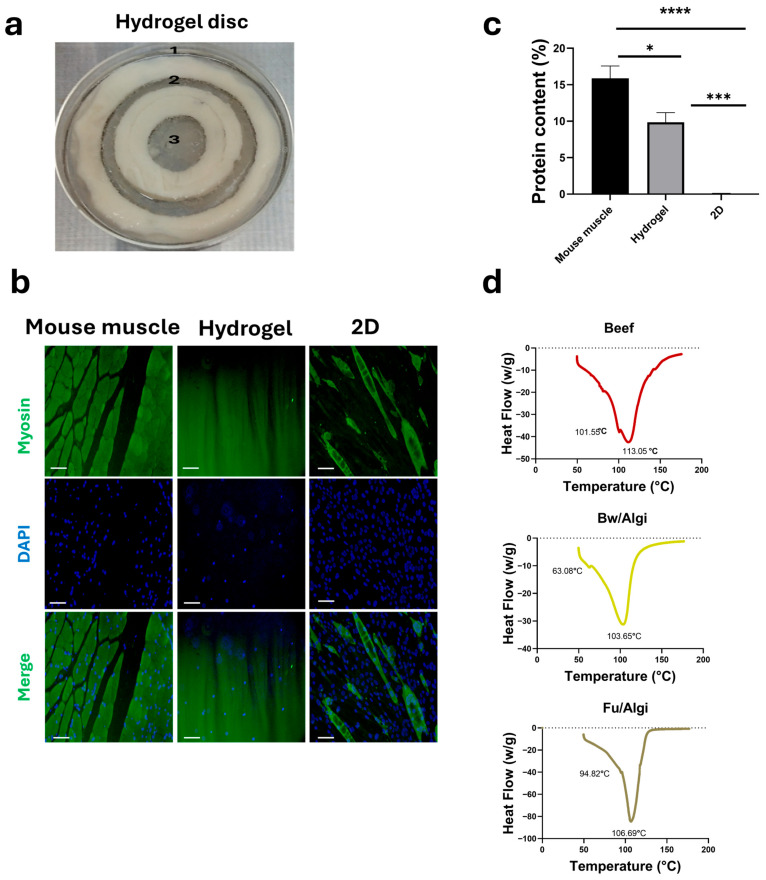
Characterization of the 3D Bw/Fu/Algi hydrogel disc: differentiation, protein accumulation, and thermal behavior. (**a**) 3D co-culture disc showing C2C12 cells (1), MEFs (2), and HECs (3) embedded in Fu/Algi hydrogel and spatially separated by a Bw/Algi at day 3 of cell growth. (**b**) Immunofluorescence staining of skeletal myosin indicating myogenic marker expression in differentiated C2C12 cells after 7 days. A 2D co-culture conditions without hydrogel and mouse muscle tissue served as controls. Scale bar = 100 μm. (**c**) Total protein content measured using a BCA assay after 10 days of differentiation. (**d**) DSC thermograms showing thermal transitions of the hydrogel components compared with native bovine muscle. Data are presented as mean ± SE (*n* = 3). Statistical significance was determined using one-way ANOVA: * *p* < 0.05, *** *p* < 0.001, and **** *p* < 0.0001.

The 2D co-culture model was first used to evaluate C2C12 growth and myogenic marker expression in the presence of MEFs, HECs, and a Bw/Algi lipid hydrogel (Figure 3). Based on the successful outcomes of the 2D system, a 3D hydrogel disc composed of Bw, Fu, and Algi was subsequently constructed to examine spatially separated cell organization (Figure 5). The 3D system included C2C12 (1), MEFs (2), and HECs (3) positioned in defined regions, while Bw/Algi functioned as a spatial barrier between cell populations. Figure 5a illustrates the compartmentalized cell distribution within the 3D hydrogel disc after 3 days of growth.

After 7 days, C2C12 cells in the 3D disc showed skeletal myosin staining with fiber-like structures (Figure 5b), indicating expression of skeletal muscle-associated protein within the hydrogel environment. Total protein content was measured after 10 days of differentiation using a BCA assay (Figure 5c), showing detectable protein accumulation in both 2D and 3D cultures. Mouse muscle contains higher protein levels than both culture systems, while the 3D hydrogel exhibited greater protein accumulation than the 2D control. The 2D condition refers to the same co-culture setup used for the 3D model without hydrogel under identical differentiation conditions. Thermal properties of the hydrogel disc were examined using DSC compared to beef tissue (Figure 5d). The hydrogel components displayed characteristic thermal transitions, demonstrating distinct thermal behavior.

Microscopy images showed that C2C12 cells in the first chamber exhibited longitudinal growth, while MEFs and HECs displayed successful growth within the Fu/Algi regions during the 10-day culture period (Figure S2a,b). CCK-8 analysis indicated cell viability of C2C12 cells, MEFs, and HECs within the 3D hydrogel disc during both proliferation and differentiation phases, compared to their respective 2D control groups (Figure S2c,d). Live/Dead staining showed the presence of viable C2C12 cells, MEFs, and HECs within the Fu/Algi regions following 20 min of crosslinking (Figure S3).

The 3D Bw/Fu/Algi construct maintained multicellular culture, enabled expression of myogenic markers, and demonstrated measurable protein accumulation together with distinct thermal behavior.

## 4. Discussion

The present work established a compartmentalized Bw/Fu/Algi hydrogel platform designed to spatially organize myoblasts, fibroblasts, and endothelial cells within a single in vitro construct for indirect multi-cell co-culture. Previous studies have shown that co-culture systems are useful for investigating coordinated cellular interactions and tissue organization [47,48]. Indirect co-culture enables the exchange of soluble biomolecules between distinct cell populations while preventing direct physical competition [48,49,50]. Such systems are particularly relevant for skeletal muscle models, where paracrine signaling plays a central role in regulating myoblast proliferation and differentiation [51].

Our previous studies focused on improving material composition and bioactivity within homogeneous hydrogel systems [5,42]. The present work introduces a compartmentalized architecture that enables the organization of multiple cell populations within a single construct. The incorporation of a Bw/Algi barrier phase allows co-culture through hydrogel separation, representing a transition from material optimization toward structural organization. This approach addresses a limitation of previous systems that did not incorporate defined multicellular spatial arrangement within a single scaffold.

Although adipose tissue contributes to meat quality, direct co-culture of adipocytes with myoblasts has been reported to suppress myogenic differentiation [9,52,53]. To address this limitation, a plant-derived Bw/Algi hydrogel was used as a lipid-containing barrier rather than incorporating live adipocytes. In parallel, Fu/Algi hydrogels were used as the primary cell-laden compartments. This material combination enabled the growth of different cell populations while preserving indirect communication, within a single scaffold.

Co-culturing more than two cell types remains challenging due to differences in growth behavior and signaling dynamics [49]. Monoculture systems fail to recapitulate the complexity of native muscle tissue [54,55,56]. Fibroblasts contribute to extracellular matrix organization, while endothelial cells provide trophic signals that support cell survival and tissue development [54,57]. In the present study, the inclusion of MEFs and HECs maintained an indirect configuration without interfering with myogenic progression, consistent with previous reports demonstrating the benefits of multicellular systems over monocultures [57,58].

The 2D co-culture system provided a controllable platform to assess indirect interactions among C2C12 cells, MEFs, HECs, and the Bw/Algi biomaterials. This configuration enabled conditioned media exchange while maintaining compartmental organization. C2C12 cells maintained proliferative activity and expressed myogenic markers under these conditions. Although endothelial cells alone have been reported to suppress myogenic differentiation [13,59,60], the combined presence of fibroblasts and the Bw/Algi barrier did not adversely affect proliferation or differentiation marker expression. Protein accumulation further suggests that indirect signaling during the proliferation phase supported subsequent differentiation [61].

Beeswax alone has limited applicability in tissue engineering due to restricted porosity and permeability [62,63]. In this study, blending Bw with alginate increased pore area and improved scaffold porosity while preserving characteristic chemical features. The reduced swelling of Bw/Algi compared with alginate alone is consistent with the hydrophobic nature of lipid-containing composites [64]. Importantly, the composite scaffold remained cytocompatible and supported initial cell attachment, supporting its function as a barrier phase rather than a primary cell-laden matrix.

Although SEM indicated a porous surface morphology and the Bw/Algi hydrogel exhibited swelling behavior, these observations do not provide direct information regarding cell passage across the barrier. In this system, the Bw/Algi layer was introduced to separate neighboring cell-laden regions and enable co-culture within the three-dimensional construct. Qualitative analysis of DAPI-stained sections showed limited cell presence at the central barrier during proliferation, no visible cells at the peripheral barrier, and no visible cells at either barrier after differentiation. These observations indicate that compartment separation was maintained under the tested conditions. However, molecular transport across the barrier was not evaluated in this study and requires further investigation.

Such structural heterogeneity may influence transport behavior within the scaffold [65]. However, diffusion of soluble factors across the Bw/Algi barrier was not evaluated in this study. Indeed, it has been widely reported that porosity alone is insufficient to predict cell infiltration without adequate pore interconnectivity [44,66]. Accordingly, although the scaffold was designed to support indirect co-culture, regulated molecular exchange across the barrier was not mechanistically verified.

While alginate enables mild ionic gelation and straightforward scaffold fabrication, its limited intrinsic bioactivity can restrict efficient myogenic development without additional functional components. [8,67]. The incorporation of Fu enhanced scaffold bioactivity, supporting cell viability and myogenic marker expression [68,69,70]. However, the observed results should be interpreted as early-stage myogenic progression. Additional assessments, including myosin heavy chain (MyHC), sarcomeric organization, contractility, and calcium handling, were not performed; therefore, the extent of myogenic maturation remains to be further evaluated.

Within the 3D construct, C2C12 cells expressed skeletal muscle markers and accumulated detectable protein during differentiation. Although this design does not replicate full tissue maturation or vascularization, it provides a reproducible platform for evaluating cell co-culture relevant to cultured meat scaffold development [71,72].

Cells within the 3D construct were exposed to CaCl_2_ during hydrogel crosslinking, which may influence intracellular signaling and differentiation processes. Although immediate post-crosslinking viability was confirmed by Live/Dead staining, the potential effects of calcium exposure on cellular signaling were not directly evaluated in this study.

The experimental design was based on a stepwise evaluation of material composition, co-culture configuration, and 3D architecture. However, not all control conditions required to fully isolate the individual contributions of Fu, Bw, co-cultured cells, and dimensional context were included. Therefore, additional matched controls are required to delineate the specific role of each component.

While equal cell numbers were seeded across compartments of different volumes, this study primarily aimed to establish a spatially co-culture platform rather than to evaluate density-dependent cellular responses. Accordingly, the effect of local cell density variation was not assessed and remains a limitation of the current study.

Thermal stability is an important consideration for edible scaffolds intended for downstream processing. Heating influences protein structure and meat-like textural properties [73,74,75]. DSC measurements exceeded typical conditions for protein denaturation analysis. Accordingly, thermal transitions may shift and broaden due to thermal lag, and the data are interpreted comparatively rather than as absolute values.

Murine muscle tissue was used as a reference for myosin immunofluorescence and total protein quantification to align with the C2C12 cell model, whereas native bovine muscle (beef) was used for DSC analysis due to its relevance to food-related thermal properties. These comparisons are intended as contextual benchmarks rather than direct cross-species equivalence.

The material characterization in this study was limited to physicochemical properties, swelling and degradation behavior, cytocompatibility, and thermal analysis. Mechanical properties, including compressive modulus, as well as rheological and viscoelastic behavior, were not evaluated. In addition, diffusion-related properties relevant to nutrient and factor transport across the scaffold were not assessed. Therefore, the material performance of the Bw/Fu/Algi system should be interpreted within the scope of these initial assessments.

This employs a mixed-species co-culture system combining murine (C2C12 and MEF) and human (EA.hy926) cell lines. As previously reported, species-specific differences in receptor–ligand interactions may influence the magnitude and specificity of signaling responses [76]. Accordingly, the observed outcomes should be interpreted as general co-culture patterns rather than precise biological responses. Future studies will be necessary to indicate the accuracy of intercellular signaling analysis and enhance translational relevance.

The observed biodegradation indicates partial mass loss over time; however, these measurements do not provide information regarding structural integrity, which depends on mechanical properties and network architecture. Therefore, this system should be considered a preliminary platform for multicellular scaffold design rather than a fully developed food-grade cultured meat system. Although region-specific imaging provides preliminary observations, it does not verify long-term compartmental or structural integrity. Accordingly, future studies incorporating cell-tracking approaches, as well as mechanical and structural analyses, are required to comprehensively assess scaffold behavior over time.

## 5. Conclusions

This study establishes a compartmentalized in vitro co-culture platform integrating C2C12 myoblasts, fibroblasts, and endothelial cells within a Bw/Fu/Algi composite hydrogel. The system supported cell viability and early-stage myogenic differentiation under 3D culture conditions. Translation of the 2D quadrate design into a 3D disc architecture enabled indirect tri-culture while maintaining compartmental separation, protein accumulation, and thermal scaffold stability. Collectively, these results demonstrate the feasibility of lipid-containing and polysaccharide-based composite hydrogels as a proof-of-concept platform for structured multi-cell-type in vitro culture. Although full tissue maturation and equivalence to beef muscle were not achieved, the platform provides a modular foundation for scaffold design relevant to cultured meat research. Further studies incorporating livestock-derived cells and comprehensive food-grade evaluations will be necessary to enhance translational relevance and scalability.

## Figures and Tables

**Table 1:** Murine primers used in real-time RT–qPCR analysis.

Gene	Primer Sequence (5′-3′)
*GAPDH*	F: CATCACTGCCACCCAGAAGACTG R: ATGCCAGTGCTTCCCGTTCAG
*β-actin*	F: GAAATCGTGCGTGACATCAAA R: TGTAGTTTCATGGATGCCACA
*Cdk1*	F: TTGAGAGTGTGAGGCAGGAG R: TCCCAAGGAGTAGGCTAGGT
*Myogenin*	F: AGAGACATGAGTGCCCTGAC R: TTCCCGGTATCATCAGCACA

## Data Availability

The original contributions presented in this study are included in the article and Supplementary Materials. Further inquiries can be directed to the corresponding author.

## Supplementary Materials

The following supporting information can be downloaded at: https://www.mdpi.com/article/10.3390/foods15101715/s1, Figure S1. Biodegradability of the Bw/Algi barrier in a 3D co-culture hydrogel system. (a) Biodegradability of the Bw/Algi barrier during proliferation and (b) differentiation (*n* = 4). Figure S2. 3D co-culture hydrogel disc during proliferation and differentiation. (a)&(b) Three-dimensional (3D) co-culture hydrogel disc showing proliferation of C2C12 cells, MEFs, and HECs within Fu/Algi regions surrounded by a Bw/Algi barrier over 10 days (3 days proliferation and 7 days differentiation). Scale bar = 200 μm. (c) and (d) CCK-8 assay was performed on cells cultured in Fu/Algi within the 3D disc during both proliferation and differentiation periods (*n* = 5). The control group represents a 2D culture for each cell type. Figure S3. Three-dimensional (3D) co-culture hydrogel disc showing C2C12 cells, MEFs, and HECs within Fu/Algi regions surrounded by a Bw/Algi barrier. Fu/Algi-containing regions in each chamber were punched using a 6 mm punch. Live/dead staining was performed 20 minutes after crosslinking with CaCl_2_. Scale bar = 200 μm.

## Abbreviations

Algi	Alginate
Bw	Beeswax
Bw/Algi	Beeswax/alginate
Fu	Fucoidan
Fu/Algi	Fucoidan-alginate
MEF	Mouse embryonic fibroblast
HECs	Human endothelial cells
FI (%)	Fusion Index percentage
