# Supplementary material for: Engineering a Compartmentalized Multi-Cell Co-Culture Hydrogel System Using Beeswax/Fucoidan/Alginate for Cultured Meat Modeling
Source: Foods. 2026 May 13;15(10):1715. doi: 10.3390/foods15101715 (PMC13206685; doi:10.3390/foods15101715)
Supplement: Supplementary file 1 [file foods-15-01715-s001.zip › foods-4214779-supplementary.pdf]

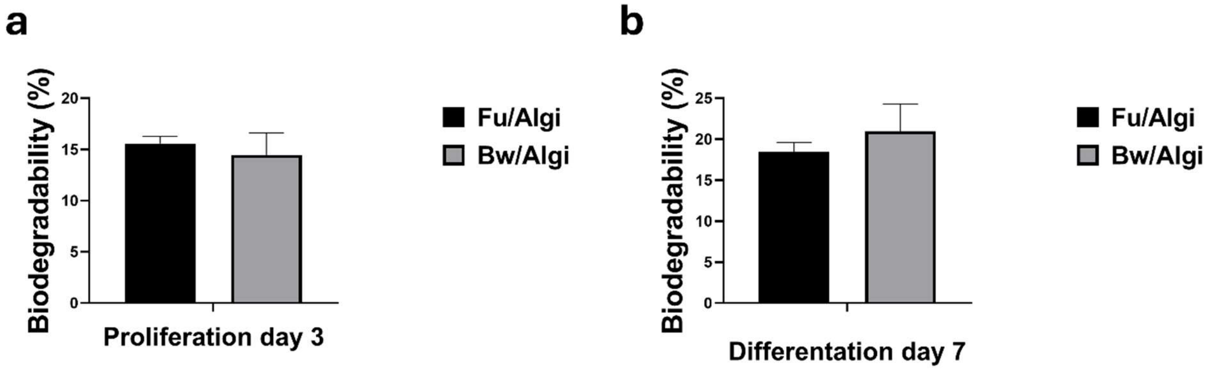

Figure S1. Biodegradability of the Bw/Algi barrier in a 3D co-culture hydrogel system. (a) Biodegradability of the Bw/Algi barrier during proliferation and (b) differentiation (n = 4).

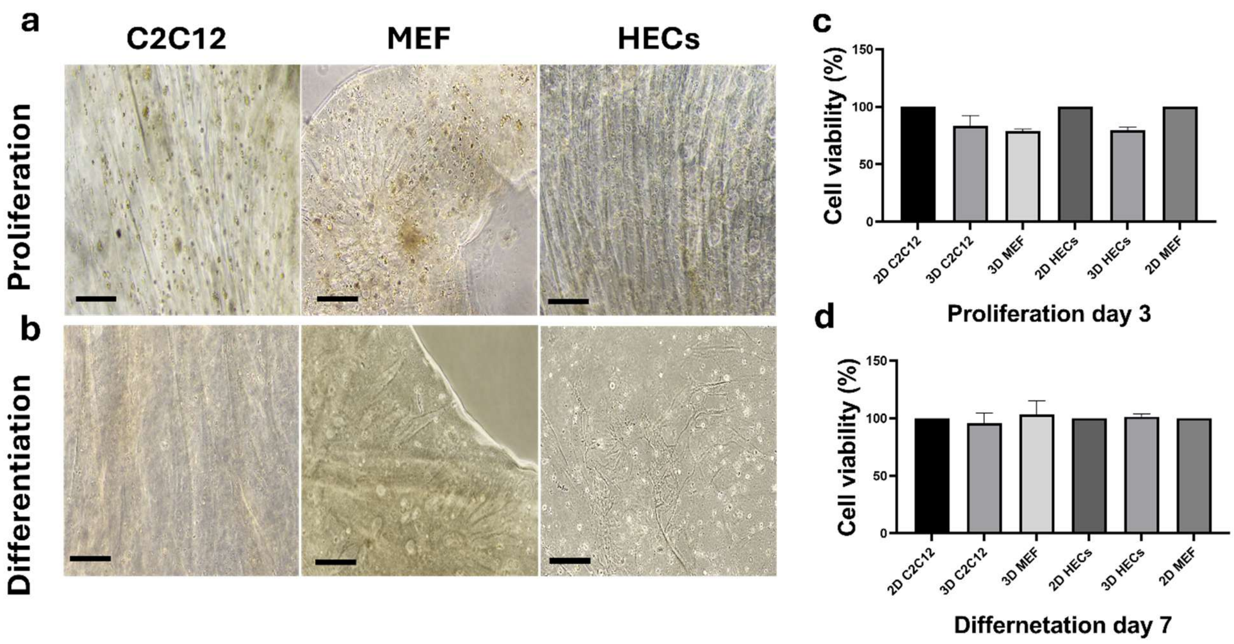

Figure S2. 3D co-culture hydrogel disc during proliferation and differentiation. (a)&(b) Three-dimensional (3D) co-culture hydrogel disc showing proliferation of C2C12 cells, MEFs, and HECs within Fu/Algi regions surrounded by a Bw/Algi barrier over 10 days (3 days proliferation and 7 days differentiation). Scale bar = 200 µm. (c) and (d) CCK-8 assay was performed on cells cultured in Fu/Algi within the 3D disc during both proliferation and differentiation periods (n = 5). The control group represents a 2D culture for each cell type.

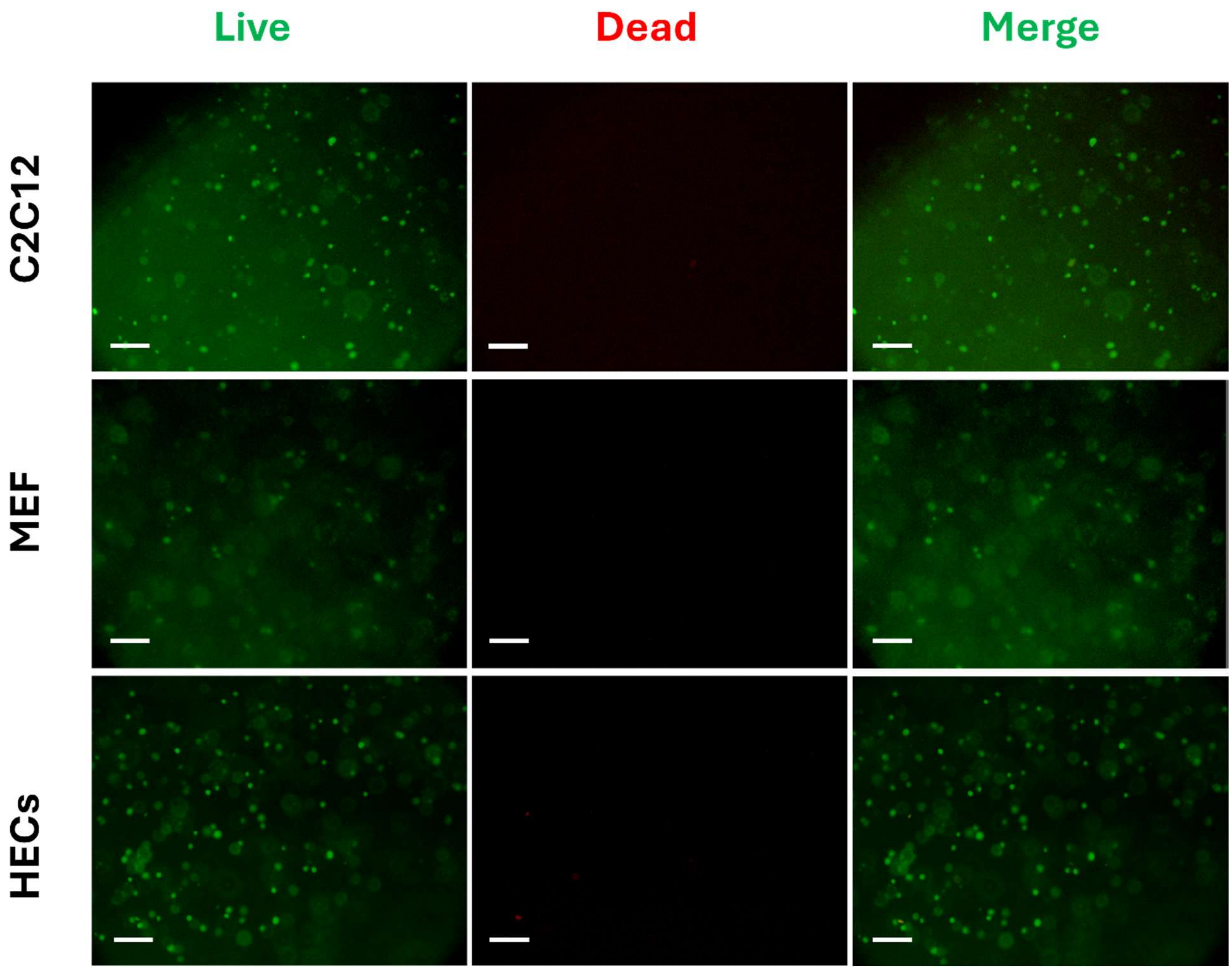

Figure S3. Three-dimensional (3D) co-culture hydrogel disc showing C2C12 cells, MEFs, and HECs within Fu/Algi regions surrounded by a Bw/Algi barrier. Fu/Algi-containing regions in each chamber were punched using a 6 mm punch. Live/dead staining was performed 20 minutes after crosslinking with $CaCl_2$. Scale bar = 200 μm.
